# Supplementary material for: First Report and Comparative Genomic Analysis of Mycoplasma capricolum subsp. capricolum HN-B in Hainan Island, China
Source: Microorganisms. 2022 Nov 19;10(11):2298. doi: 10.3390/microorganisms10112298 (PMC9697796; doi:10.3390/microorganisms10112298)
Supplement: Supplementary file 1 [file microorganisms-10-02298-s001.zip › Figure S1 Variation type display diagram.pdf]

Figure S1. Variation type display diagram

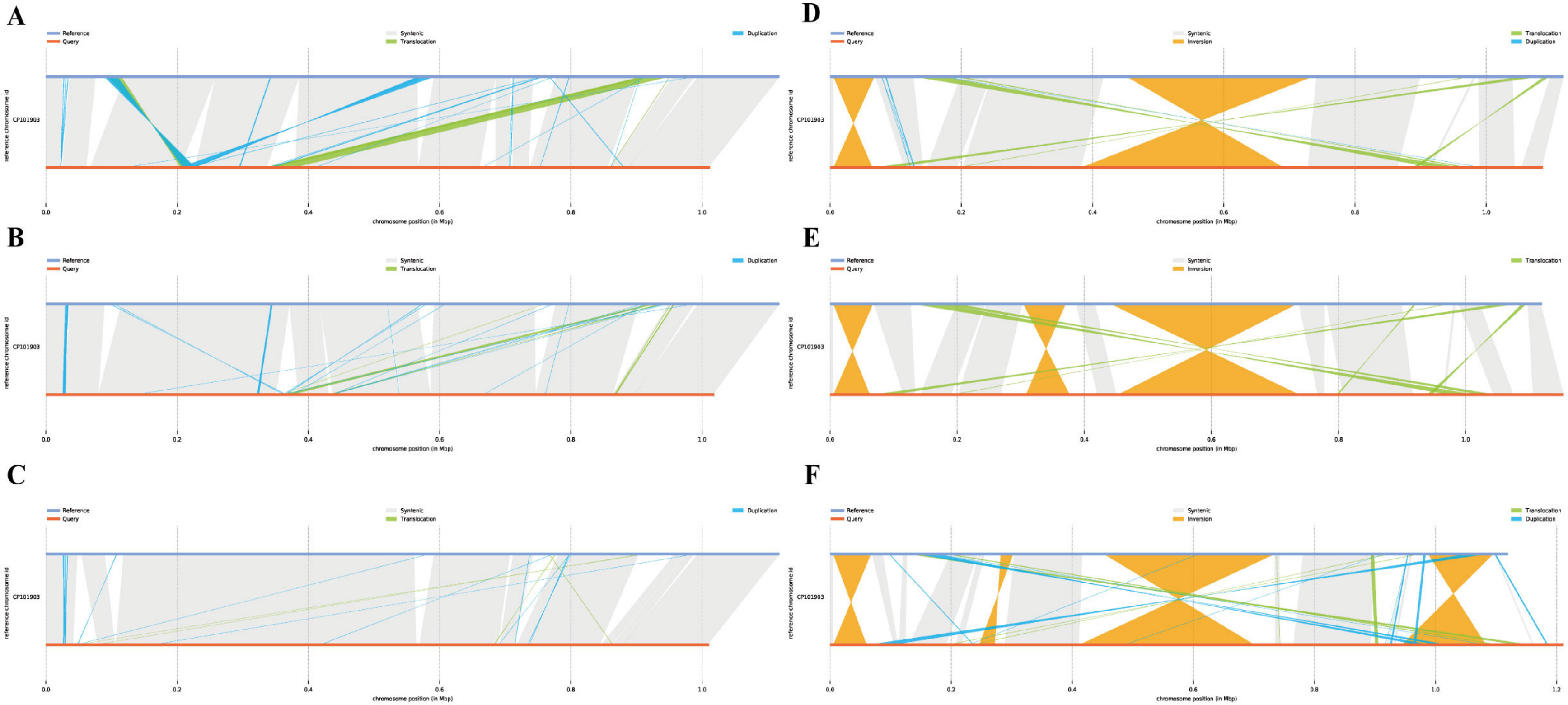

Figure S1. Variation type display diagram

(A) Variation type display diagram of Mcc ATCC 2734 vs Mcc HN-B

(B) Variation type display diagram of Mccp M1601 vs Mcc HN-B

(C) Variation type display diagram of Ml PG50 vs Mcc HN-B

(D) Variation type display diagram of Mmc HN-A vs Mcc HN-B

(E) Variation type display diagram of MmmLC 95010 vs Mcc HN-B

(F) Variation type display diagram of MmmSC PG1 vs Mcc HN-B
